# 

**Published:** 1855-04-01

**Authors:** 


Letters and Books for the Editor may be transmitted either through the Publisher or direct to Dr. Winslow, 23, Cavendish-square, London.

